# Supplementary material for: High sugar diets can increase susceptibility to bacterial infection in Drosophila melanogaster
Source: PLoS Pathog. 2024 Aug 12;20(8):e1012447. doi: 10.1371/journal.ppat.1012447 (PMC11341100; doi:10.1371/journal.ppat.1012447)
Supplement: S1 Fig — (A) There was no effect of diet on survivorship after infection with Enterococcus faecalis at an inoculation dose of ~1000 colony forming units (CFU) per host. (B) Flies fed the 16% sucrose diet have significantly higher mortality than flies fed 2% sucrose with a higher infection dose (~3000 CFU/fly) of E. faecalis (p = 0.01; Cox Mixed Effects Model), while there was still no effect of diet on survivorship with E. faecalis in a replicate experiment at an inoculum of ~1000 CFUs (p = 0.14). Dashed lines indicate a lower infection dose (~1000 CFU/fly) and solid lines indicate a higher inoculation dose (~3000 CFU/fly). (C) Flies fed the 24% sucrose diet have the highest mortality after Lactococcus lactis infection compared to all other diets at an inoculation dose of ~1000 CFU/fly (p<0.001, Cox Mixed Effects Model). Flies fed 16% sucrose died significantly faster than flies fed 0% sucrose diets (p = 0.047; Cox Mixed Effects Model), but there were no other significant pairwise differences in survivorship among flies provided the 16% sucrose diet or lower. Letters denote significant pairwise differences between diets (p<0.05). (D) Flies fed the 16% sucrose diet have significantly higher mortality than flies fed 2% sucrose after L. lactis infection with ~1000 CFU/fly (p< 0.001; Cox Mixed Effects Model) or ~3000 CFU/fly infection doses (p < 0.001; Cox Mixed Effects Model). Dashed lines indicate a lower infection dose (~1000 CFU/fly) and solid lines indicate a higher inoculation dose (~3000 CFU/fly). (DOCX) [file ppat.1012447.s001.docx]

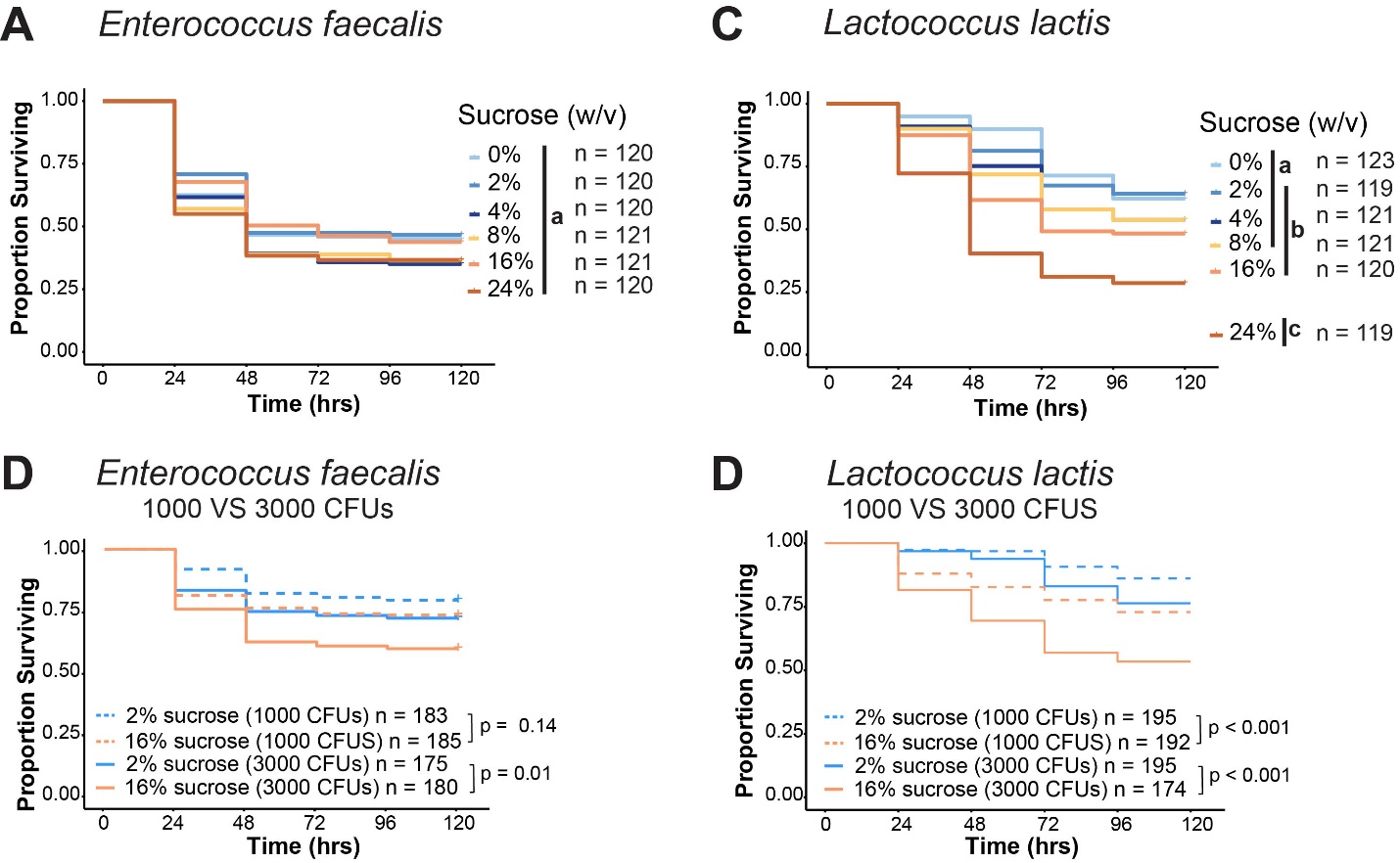


**S1 Fig.** Impact of high-sugar diets on survival of infection with Gram-positive bacteria. (A) There was no effect of diet on survivorship after infection with *Enterococcus faecalis* at an inoculation dose of ~1000 colony forming units (CFU) per host. (B) Flies fed the 16% sucrose diet have significantly higher mortality than flies fed 2% sucrose with a higher infection dose (~3000 CFU/fly) of *E. faecalis* (p = 0.01; Cox Mixed Effects Model), while there was still no effect of diet on survivorship with *E. faecalis* in a replicate experiment at an inoculum of ~1000 CFUs (p = 0.14). Dashed lines indicate a lower infection dose (~1000 CFU/fly) and solid lines indicate a higher inoculation dose (~3000 CFU/fly). (C) Flies fed the 24% sucrose diet have the highest mortality after *Lactococcus lactis* infection compared to all other diets at an inoculation dose of ~1000 CFU/fly (p<0.001, Cox Mixed Effects Model). Flies fed 16% sucrose died significantly faster than flies fed 0% sucrose diets (p = 0.047; Cox Mixed Effects Model), but there were no other significant pairwise differences in survivorship among flies provided the 16% sucrose diet or lower. Letters denote significant pairwise differences between diets (p<0.05). (D) Flies fed the 16% sucrose diet have significantly higher mortality than flies fed 2% sucrose after *L. lactis* infection with ~1000 CFU/fly (p< 0.001; Cox Mixed Effects Model) or ~3000 CFU/fly infection doses (p < 0.001; Cox Mixed Effects Model). Dashed lines indicate a lower infection dose (~1000 CFU/fly) and solid lines indicate a higher inoculation dose (~3000 CFU/fly).
