# Supplementary material for: High sugar diets can increase susceptibility to bacterial infection in Drosophila melanogaster
Source: PLoS Pathog. 2024 Aug 12;20(8):e1012447. doi: 10.1371/journal.ppat.1012447 (PMC11341100; doi:10.1371/journal.ppat.1012447)

**S2 Fig.** A) Survivorship of PBS injury controls across all infection experiments pooled together. Flies fed 24% (w/v) sucrose diet have higher proportion of death after sham-infection with sterile PBS compared to all other diets (p<0.05; Cox Proportional Hazards Mixed effects model).


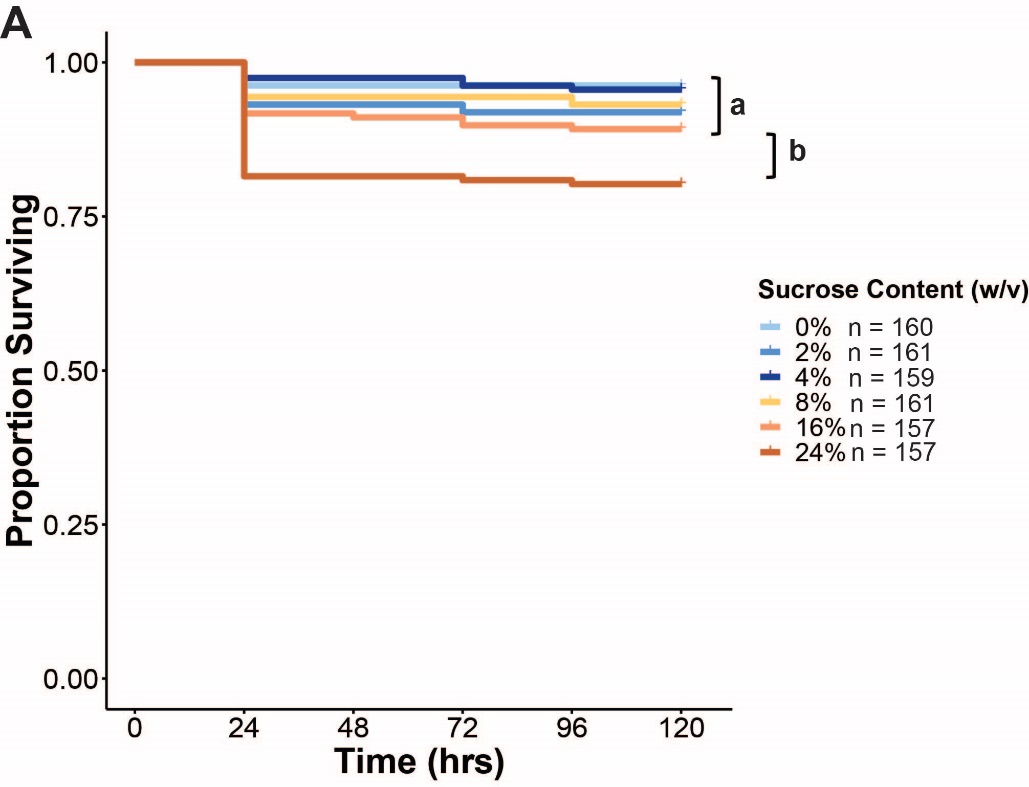

Supplement: S2 Fig — A) Survivorship of PBS injury controls across all infection experiments pooled together. Flies fed 24% (w/v) sucrose diet have higher proportion of death after sham-infection with sterile PBS compared to all other diets (p<0.05; Cox Proportional Hazards Mixed effects model). (DOCX) [file ppat.1012447.s002.docx]
