# Supplementary material for: High sugar diets can increase susceptibility to bacterial infection in Drosophila melanogaster
Source: PLoS Pathog. 2024 Aug 12;20(8):e1012447. doi: 10.1371/journal.ppat.1012447 (PMC11341100; doi:10.1371/journal.ppat.1012447)

**S3 Fig.** A) To measure the abundance of microbiota found in uninfected flies fed on the 2% (w/v) sucrose or 16% sucrose diets, 10 whole flies were pooled and homogenized in 250 μl of PBS, and 50 μl of the homogenate was plated on lysogeny broth (LB) or brain heart infusion (BHI) agar. Flies fed on the 2% diets exhibit higher microbiota loads on LB (p<0.001, n = 15 pools of 10 flies per diet) and BHI (p<0.001, n = 15 pools of 10 flies per diet) plates than flies fed 16% sucrose. B) Axenic flies were infected with *P. rettgeri*, and flies fed 16% sucrose (n = 78) diets exhibited higher mortality than flies given 2% sucrose (n = 76; p<0.001; Cox Proportional Hazards model).


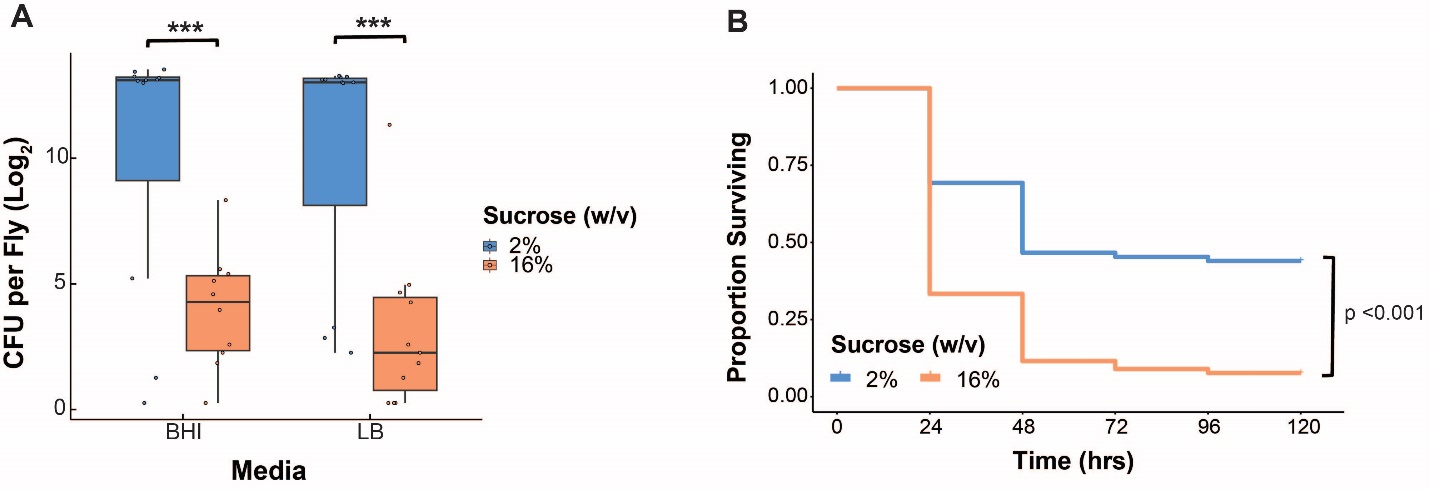

Supplement: S3 Fig — A) To measure the abundance of microbiota found in uninfected flies fed on the 2% (w/v) sucrose or 16% sucrose diets, 10 whole flies were pooled and homogenized in 250 μl of PBS, and 50 μl of the homogenate was plated on lysogeny broth (LB) or brain heart infusion (BHI) agar. Flies fed on the 2% diets exhibit higher microbiota loads on LB (p<0.001, n = 15 pools of 10 flies per diet) and BHI (p<0.001, n = 15 pools of 10 flies per diet) plates than flies fed 16% sucrose. B) Axenic flies were infected with P. rettgeri, and flies fed 16% sucrose (n = 78) diets exhibited higher mortality than flies given 2% sucrose (n = 76; p<0.001; Cox Proportional Hazards model). (DOCX) [file ppat.1012447.s003.docx]
