# Supplementary material for: High sugar diets can increase susceptibility to bacterial infection in Drosophila melanogaster
Source: PLoS Pathog. 2024 Aug 12;20(8):e1012447. doi: 10.1371/journal.ppat.1012447 (PMC11341100; doi:10.1371/journal.ppat.1012447)

**S4 Fig.** Soluble protein levels across diet and infection conditions were used to normalize carbohydrate levels. Flies were pooled in batches of 3 for each biological replicate. Protein levels were ~10% lower in flies fed 16% sucrose compared to flies fed 2% sucrose across all infection treatments (uninfected, p = 0.013, n = 30 ; *Providencia rettgeri,* p = 0.013, n= 25, *Serratia marcescens,* p = 0.014, n = 30; post-hoc Tukey test p<0.05). Legend for panel figure: * = p<0.05


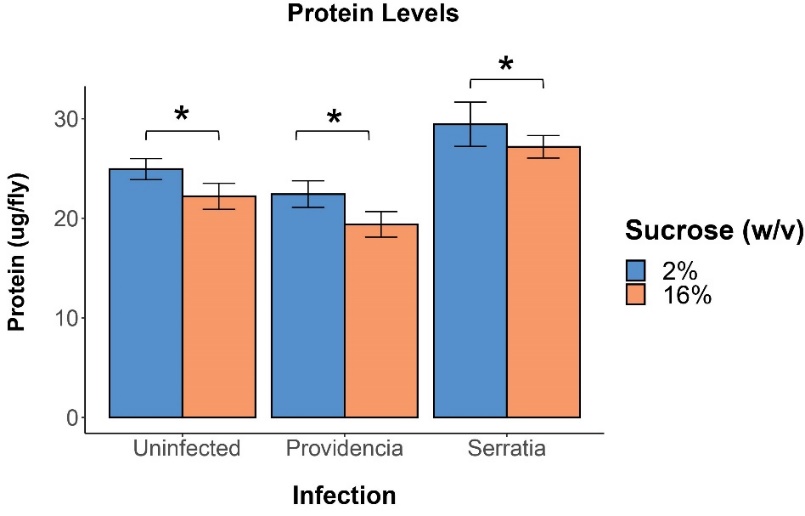

Supplement: S4 Fig — Flies were pooled in batches of 3 for each biological replicate. Protein levels were ~ 10% lower in flies fed 16% sucrose compared to flies fed 2% sucrose across all infection treatments (uninfected, p = 0.013, n = 30; Providencia rettgeri, p = 0.013, n = 25, Serratia marcescens, p = 0.014, n = 30; post-hoc Tukey test p<0.05). Legend for panel figure: * = p<0.05 (DOCX) [file ppat.1012447.s004.docx]
