# Supplementary material for: High sugar diets can increase susceptibility to bacterial infection in Drosophila melanogaster
Source: PLoS Pathog. 2024 Aug 12;20(8):e1012447. doi: 10.1371/journal.ppat.1012447 (PMC11341100; doi:10.1371/journal.ppat.1012447)

**S5 Fig.** We compared pathogen growth within wildtype Canton S (CS) and ΔAMP10 mutant flies using data from Figs 3A-B and Figs 4A-B to visualize how pathogen growth differs between the genotypes on the 2% (A, B) and 16% (C, D) diets. Pathogen loads from CS flies were plotted until the last time point with surviving ΔAMP10 mutants, which was 8-hours for *S. marcescens* infection (A,C) and 12-hours for *P. rettgeri* infection (B,D). (A-D) Across all diet and infection contrasts, ΔAMP10 mutants exhibit significantly higher bacterial loads compared to wildtype CS in the last three hours of the time course (p<0.05; GLS model). Each time point contains sample sizes ranging from 8-28 individual flies. Legend for panel figure: * = p<0.05, ** = p<0.01, *** = p <0.001.


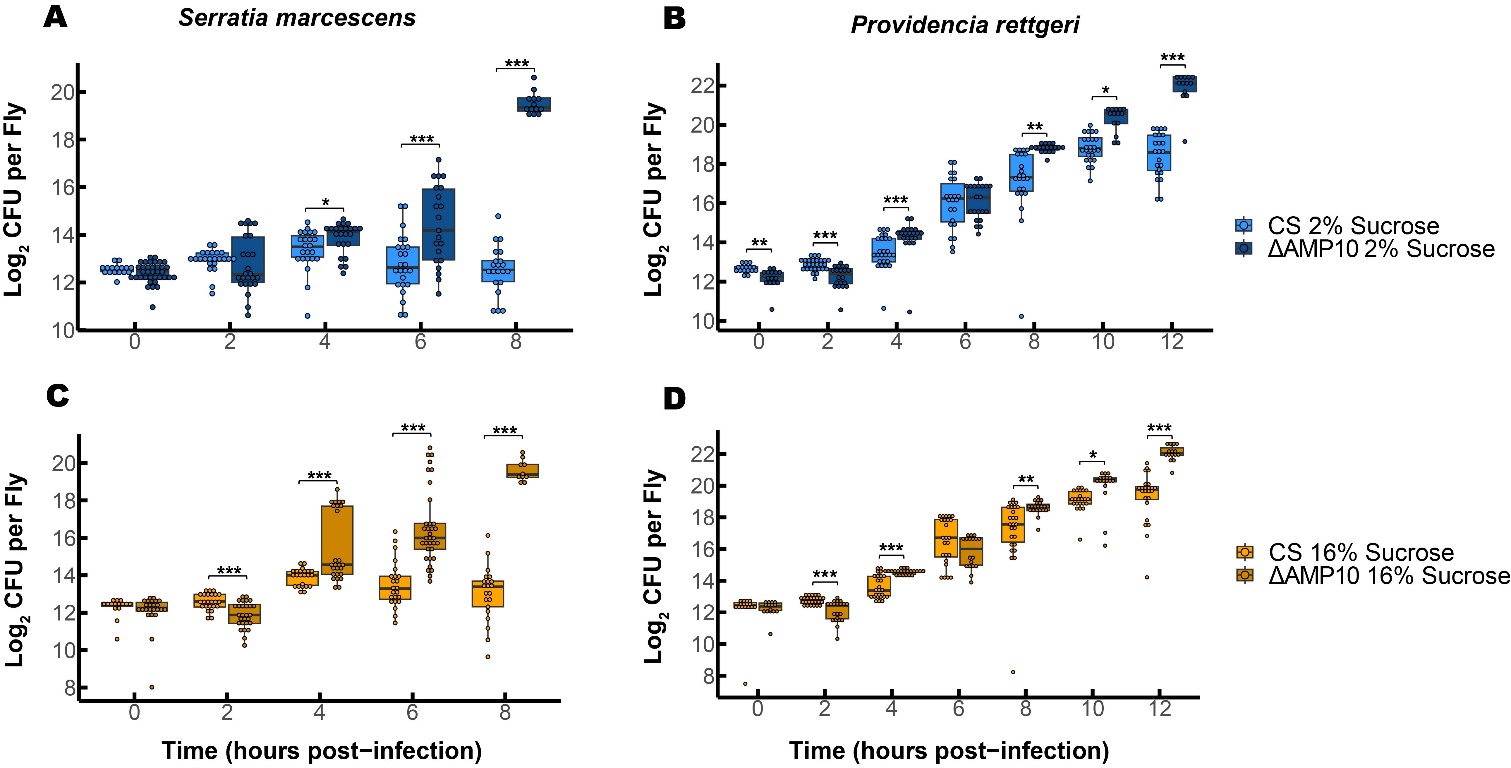

Supplement: S5 Fig — We compared pathogen growth within wildtype Canton S (CS) and ΔAMP10 mutant flies using data from Figs 3A, 3B, 4A and 4B to visualize how pathogen growth differs between the genotypes on the 2% (A, B) and 16% (C, D) diets. Pathogen loads from CS flies were plotted until the last available time point of live ΔAMP10 mutants, which was 8-hours for S. marcescens infection (A,C) and 12-hours for P. rettgeri infection (B,D). (A-D) Across all diet and infection contrasts, ΔAMP10 mutants exhibit significantly higher bacterial loads compared to wildtype CS in the last three hours of the time course (p<0.05; GLS model). Each time point contains sample sizes ranging from 8–28 individual flies. Legend for panel figure: * = p<0.05, ** = p<0.01, *** = p <0.001. (DOCX) [file ppat.1012447.s005.docx]
