# Supplementary material for: High sugar diets can increase susceptibility to bacterial infection in Drosophila melanogaster
Source: PLoS Pathog. 2024 Aug 12;20(8):e1012447. doi: 10.1371/journal.ppat.1012447 (PMC11341100; doi:10.1371/journal.ppat.1012447)
Supplement: S6 Fig — (A) Tagged Drosocin levels are significantly higher 3 hours after injection with PBS (n = 8; p = 0.0455) or ~6.0x10^4^ cells of the Gram-negative bacterium Enterobacter cloacae (n = 8; p<0.001) than they are in uninjured flies (n = 8). E. cloacae infection results in significantly higher Drosocin levels than PBS injection (n = 11: p<0.001; linear model with Tukey post-hoc). (B) Tagged Cecropin levels are significantly higher 3-hours after infection with E. cloacae (n = 13) than after PBS injection (n = 11; p<0.001) or in uninjured flies (n = 8; p<0.001). There was no detectable difference in Cecropin levels between uninjured and PBS-injected flies (p = 0.32). Letters denote pairwise difference p<0.05. (DOCX) [file ppat.1012447.s006.docx]

**S6 Fig.** Demonstration that HA- and FLAG-tagged Drosocin and Cecropin A1 can be detected by sandwich ELISA following bacterial infection. (A) Tagged Drosocin levels are significantly higher 3 hours after injection with PBS (n = 8; p = 0.0455) or ~6.0x10^4^ cells of the Gram-negative bacterium *Enterobacter cloacae* (n = 8; p<0.001) than they are in uninjured flies (n= 8). *E. cloacae* infection results in significantly higher Drosocin levels than PBS injection (n = 11: p<0.001; linear model with Tukey post-hoc). (B) Tagged Cecropin levels are significantly higher 3-hours after infection with *E. cloacae* (n = 13) than after PBS injection (n = 11; p<0.001) or in uninjured flies (n = 8; p<0.001). There was no detectable difference in Cecropin levels between uninjured and PBS-injected flies (p = 0.32). Letters denote pairwise difference p<0.05.

**
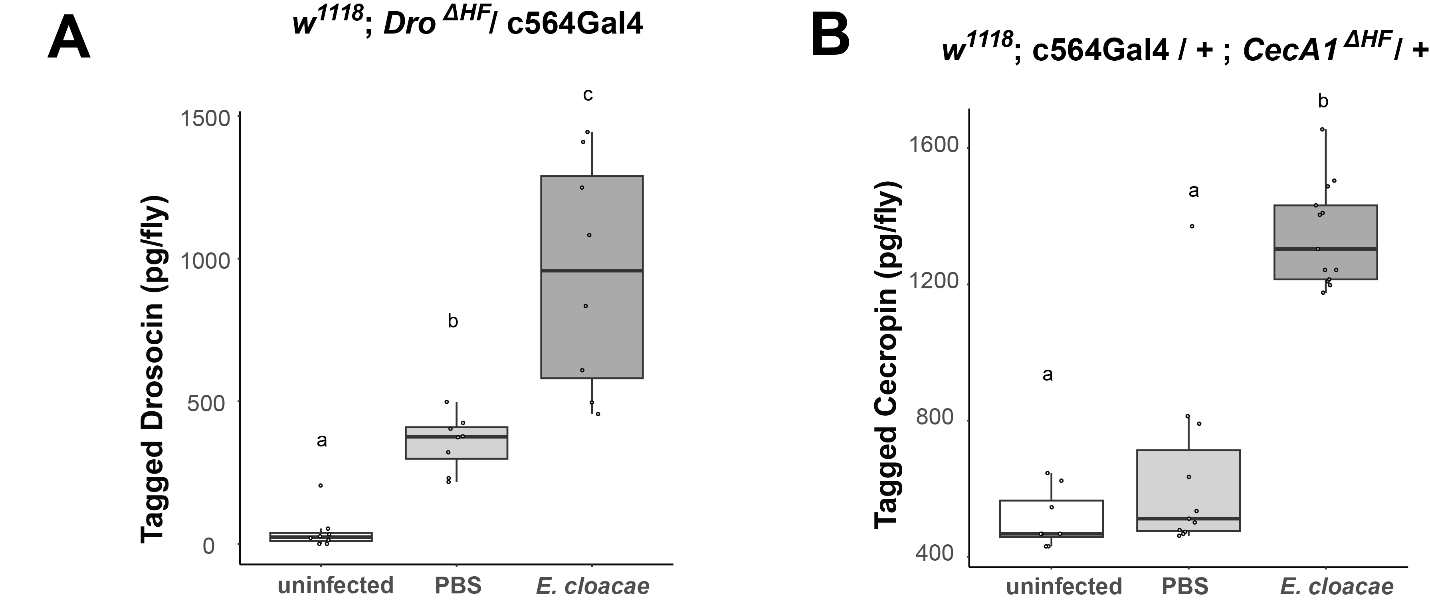
**
