# Supplementary material for: High sugar diets can increase susceptibility to bacterial infection in Drosophila melanogaster
Source: PLoS Pathog. 2024 Aug 12;20(8):e1012447. doi: 10.1371/journal.ppat.1012447 (PMC11341100; doi:10.1371/journal.ppat.1012447)

**S7 Fig.** (A) The number of flies fed the 2% and 16% diets that have, or do not have, detectable tagged Cecropin A1 six hours after *P. rettgeri* infection. (B) The number of flies fed the 2% and 16% sucrose diets that have, or do not have, detectable tagged Cecropin six hours after *S. marcescens* infection.


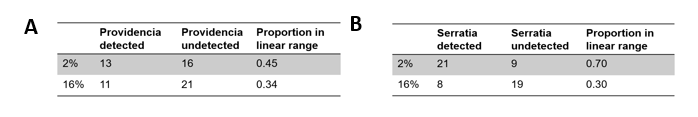

Supplement: S7 Fig — (A) The number of flies fed the 2% and 16% diets that have, or do not have, detectable tagged Cecropin A1 six hours after P. rettgeri infection. (B) The number of flies fed the 2% and 16% sucrose diets that have, or do not have, detectable tagged Cecropin six hours after S. marcescens infection. (DOCX) [file ppat.1012447.s007.docx]
