# Supplementary material for: High sugar diets can increase susceptibility to bacterial infection in Drosophila melanogaster
Source: PLoS Pathog. 2024 Aug 12;20(8):e1012447. doi: 10.1371/journal.ppat.1012447 (PMC11341100; doi:10.1371/journal.ppat.1012447)

**S8 Fig.** Newly eclosed adults were fed on the standard rearing diet for 3-days prior to transferring to the experimental diets. After 3 days of feeding on experimental diets, flies were systemically infected with (A) *S. marcescens* and (B) *P. rettgeri*. Flies fed the 16% sucrose diet exhibited significantly higher mortality after infection than flies fed the 2% sucrose diet (p<0.001; Cox mixed effects model).


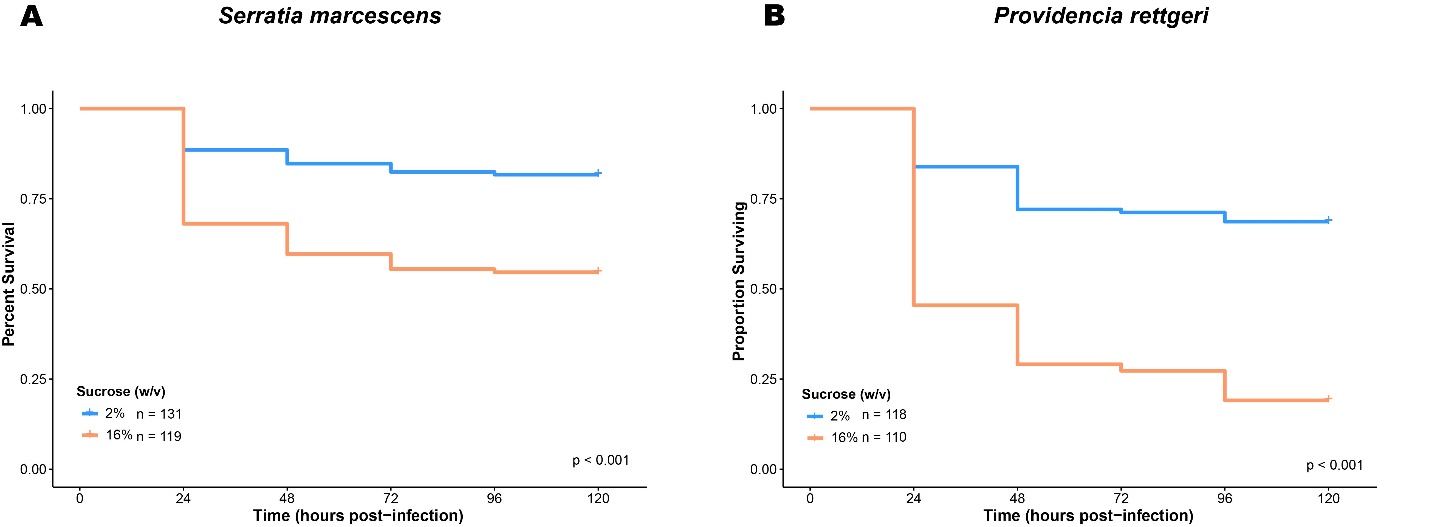

Supplement: S8 Fig — After 3 days of feeding on experimental diets, flies were systemically infected with (A) S. marcescens and (B) P. rettgeri. Flies fed the 16% sucrose diet exhibited significantly higher mortality after infection than flies fed the 2% sucrose diet (p<0.001; Cox mixed effects model). (DOCX) [file ppat.1012447.s008.docx]
