# Supplementary material for: High sugar diets can increase susceptibility to bacterial infection in Drosophila melanogaster
Source: PLoS Pathog. 2024 Aug 12;20(8):e1012447. doi: 10.1371/journal.ppat.1012447 (PMC11341100; doi:10.1371/journal.ppat.1012447)
Supplement: S1 Table — (DOCX) [file ppat.1012447.s009.docx]

**S1 Table. Primer sequences for AMP and housekeeping genes (‘5-3’).**

| **Gene** | **Forward Primer** | **Reverse Primer** |
| --- | --- | --- |
| *Actin-5C* | AGCGCGGTTACTCTTTCACCAC | GTGGCCATCTCCTGCTCA AAGT |
| *Attacin A* | CGTTTGGATCTGACCAACG | AAAGTTCCGCCAGGTGTGAC |
| *CecropinA1* | CTCTCATTCTGGCCATCACC | TCTTGAGCGATTCCCAGTC |
| *Defensin* | GCGGATCATGTCCTGGTGCAT | TCGCTTTGGCGGCTATG |
| *Diptericin A* | GCGGCGATGGTTTTGG | CGCTGGTCCACACCTTCTG |
| *Drosocin* | TTTTCCTGCTGCTTGCTTGC | GGCAGCTTGAGTCAGGTGAT |
| *Drosomysin* | CTGCCTGTCCGGAAGATACAA | TCCCTCCTCCTTGCACACA |
