# Supplementary material for: High sugar diets can increase susceptibility to bacterial infection in Drosophila melanogaster
Source: PLoS Pathog. 2024 Aug 12;20(8):e1012447. doi: 10.1371/journal.ppat.1012447 (PMC11341100; doi:10.1371/journal.ppat.1012447)

**S2 Table.** Peptide sequences, modifications, and masses of the HA-FLAG-tagged peptides and a standard used for quantification.


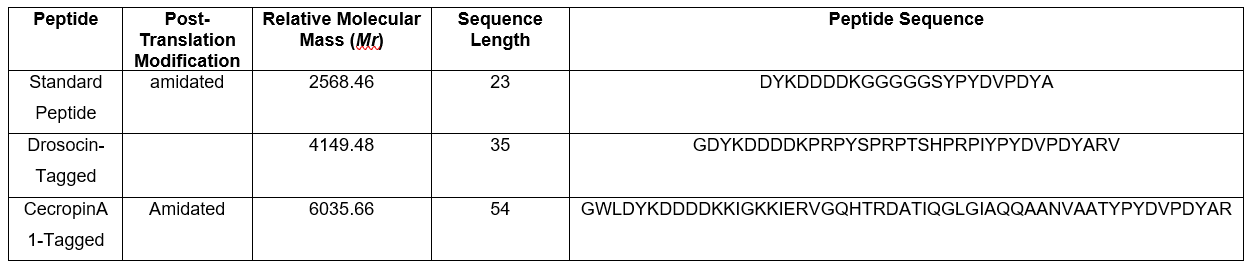

Supplement: S2 Table — (DOCX) [file ppat.1012447.s010.docx]
