# Supplementary material for: High sugar diets can increase susceptibility to bacterial infection in Drosophila melanogaster
Source: PLoS Pathog. 2024 Aug 12;20(8):e1012447. doi: 10.1371/journal.ppat.1012447 (PMC11341100; doi:10.1371/journal.ppat.1012447)
Supplement: Attachment — Submitted filename: Response_to_reviewer_15_dec_2023.pdf [file ppat.1012447.s011.pdf]

Response to Reviewer

Below is a list of revisions to the manuscript:

- Lines 41-51: Inserted an author summary for our research article as required for submission to PLOS Pathogens

- Lines 165-166: Inserted an updated version of Supplementary figure 1. There was nothing changed with the data itself. The figure letter box A previously was outlined, this version removed that outline. The survival plot width was widened, and the brackets and letters denoting statistical difference between treatments linewidth was reduced.

- Lines 168-169: Inserted an updated version of Supplementary figure 2. The data presented is the same. The outlines surrounding figure letter box A and B were removed. The outlines surrounding the boxplot and survival graph were also removed. The figure legends were edited to more accurately represent the shapes that correspond on the plot.
