# Supplementary material for: High sugar diets can increase susceptibility to bacterial infection in Drosophila melanogaster
Source: PLoS Pathog. 2024 Aug 12;20(8):e1012447. doi: 10.1371/journal.ppat.1012447 (PMC11341100; doi:10.1371/journal.ppat.1012447)
Supplement: Attachment — Submitted filename: ReviewerResponses_resubmission.docx [file ppat.1012447.s012.docx]

**Response to Review**

On behalf of the authors, I thank both reviewers for their careful reading of the paper and for their constructive feedback. We have made several adjustments to the manuscript in response to reviewer feedback, which are described in detail below, and we have additionally performed a handful of validation experiments to affirm the experimental data quality.

Reviewer comments are in blue font. Author responses are in black. Line numbers refer to the “clean” version of the manuscript without tracked changes.

**Reviewer 1:**

**Summary:**

Darby et al. investigate the effect of high-sugar diets on susceptibility to bacterial infection in Drosophila. They survey multiple bacterial species to identify two Gram-negative bacteria that increase fly mortality on high-sugar diet, and further associate sensitivity to infection with diet-induced changes to immune gene expression (at the protein level) and bacterial proliferation. The manuscript is written clearly and conservatively, and the premise for the study in relation to previous work is well described. Although there is no simple and direct take-home message here--other than the idea that diet likely impacts both flies and bacteria to affect mortality--that is probably the nature of the complex interaction between host, pathogens, and diet. The experiments are fairly well designed and executed, and the results seem solid. I've highlighted a few questions and suggestions for further study. Although additional studies would add to the completeness of the paper, it would also be reasonable to leave most of these to a follow-up study.

We appreciate the reviewer’s positive assessment of our work. We agree that there is no single take-home message, and indeed our interpretation of the results is also that diet affects infection outcome in pathogen-specific ways. We don’t see this as a shortcoming of the work, we see it as a clear-eyed assessment of the biological reality.

Responses to specific questions and suggestions are given below.

**Major Issues: Key Experiments Required for Acceptance**
I have listed all of my comments in the "minor issues" section. The only key experiments from that list of suggestions that I would really want to see are additional, controlled Delta-AMP10 lifespan and bacteria proliferation studies to better support the stated interpretations.

The bacterial proliferation assay for the delta-AMP10 flies extends until all flies have died, which occurs before 12 hours. Thus, the data shown in Figure 4 represent the entire lifespan. There is no mortality or detectable bacterial growth in uninfected flies during this period. More detailed responses on this subject are given in response to the specific comment below, and in response to a related comment by Reviewer 2.

It would also be worth checking if infected flies are able to inoculate the fly food surface with the infecting bacteria, since this is potentially a major source for a diet-bacteria interaction.

This is a thoughtful suggestion. We have done a small validation experiment, described in more detail below, and we find that environmental acquisition is not a major source of bacterial CFU in our experimental setup. Thus, we are confident in the inferences we draw from the data presented.

**Minor Issues: Editorial and Data Presentation Modifications**
METHODS: The experiments are generally clear and well designed. My greatest (still minor) concern is why the sucrose concentration series is provided immediately upon eclosion. It was my understanding that the first couple of days of adulthood (and especially the first hours post-eclosion) is still a period with some ongoing 'development' or developmental plasticity. Many studies avoid using very young adults for behavioral and other assays for this reason. I would have preferred maintaining flies on a standard diet until at least 2 days of age before starting the sucrose series. I don't think it's a huge deal here, but the authors could discuss this caveat or replicate an experiment or two with older flies.

As the reviewer suggests, there is carryover of larval fat body cells for up to 2 days into a newly eclosed adult (Aguila et al 2007, J. Exp. Biol). In principle, this could affect our experiments because the fat body is metabolically responsive to dietary nutrition and is the primary immune organ. In our experimental setup, we swapped the rearing diet for the experimental diet when the pupae formed on the chamber walls for experimental ease. We clarify that methodological point on lines 584-594. To test whether this methodology has any consequences for our main results, we performed a new infection survival experiment with adult flies that eclose on standard rearing diet and are aged for 3 days before being transferred to experimental high or low sucrose diets, where they remain for an additional 3 days before infection. We find that flies fed high sugar still have significantly higher mortality post-infection with both *S. marcescens* and *P. rettgeri* than flies fed low sugar. This additional experiment is shown in a new supplementary figure (Fig. S8).

FIG. 2: Looking at the consumption data, it seems that the difference in feeding would result in a ~400% increase in sucrose ingestion between the 2% and 16% sucrose diets used. While I agree that this is the 'bigger' change, it should be noted that protein ingestion is also reduced by about 30-35%. Thus, it is not clear if phenotypes (especially changes in AMP production) are due to more sugar, less protein, or both.

Thank you for taking the time to do the math for calculating the approximate ingestion of sucrose and protein and we agree that it is difficult to infer the difference between effects of high sugar versus low protein in voluntary-consumption experiments. Although soluble protein is not necessarily a perfect proxy for accessible protein in a biological context, we note that flies fed the high sugar diet have ~10% less soluble protein than flies fed the low sugar diet (line 187 -188), and we include a new supplementary figure S4 showing soluble protein in μg/fly.

For the metabolic indices, we state in the Methods section (line 753) that the carbohydrate nutritional indices were normalized to the amount of protein in the fly samples. We also now clarify in the Results section (lines 186-187) that protein levels were used to normalize carbohydrate levels to account for potential diet effects on body size. Although we do not observe reduced protein levels that fully reflect the reviewer’s calculated 30-35% lower protein intake, the 10% reduction in protein that we do observe could have an impact on resistance to infection, including through AMP production. We have added to the Discussion section (lines 515-518) that high-sugar diets are effectively lower in protein where we discuss possible mechanisms by which high-sugar diets might impact AMP translation, and that potentially increased mortality may be due in part to protein deficiency.

FIG. 4: The use of the Delta-AMP10 line is a great idea, but the results here are weaker. In Fig. 4A, diet does not affect P. rettgeri proliferation in the fly mutant. But only time points up to 12 hours are shown. In Fig. 3A, there is also no difference in P. rettgeri proliferation in Canton-S until 12 hours and later. It would thus be better to include later time points for the Delta-AMP10 study (as well as its genetic background control assayed in the same trial). The results for S. marcescens are even more challenging to interpret, since 16% sugar seems to consistently reduce bacterial load early, then increase it later, but only for a few hours, after which they're equivalent until much later. I agree though that, at least early in the infection, the effect of diet on S. marcescens seems reasonably consistent between genotypes. Also, what does survival look like in Delta-AMP10 upon infection? That could go a long way in supporting some of the interpretations of the proliferation data.

The experiment illustrated in Figure 4 extends until all Delta-AMP10 (dAMP10) flies have died. Because these flies are extremely susceptible to infection, all succumb by 8 hours after infection with *Serratia* and 12 hours after infection with *Providencia*. Thus, there are no later time points to examine. We now clarify this on lines 282-285.

We have added a new Supplementary Figure 5 (referenced on lines 288-289) that compares bacterial burden from the dAMP10 mutants in Fig. 4A-B to the wildtype CS flies in Fig 3A-B on the 2% and 16% diets, showing much higher burdens in the mutants. While this cannot be a directly tested comparison because the infections were performed separately, the figure provides a strong visual illustration that the higher pathogen loads of the dAMP10 mutants relative to the wildtype. On lines 289-291, we note that the high pathogen burden carried by the immunocompromised flies at the later time points reflects the lethal bacterial burden.

We understand the reviewer’s desire to have a matched background control for the dAMP10 strain. However, this line was generated by intercrossing seven different mutant lines that lacked subsets of ten inducible AMPs (Hanson et al 2019, eLife.44341) and therefore no appropriate genetic background control exists. We believe the host mortality and bacterial growth phenotypes are so striking that it seems inconceivable that they could be due to factors in the genetic background as opposed to the known AMP deficiency.

We agree that the Serratia proliferation result in the dAMP10 mutant requires interpretation. We now include further discussion of these data on lines 492-499 when we first introduce the observation of lower pathogen load at 2 hrs for both genotypes. We emphasize the higher pathogen load at hours 4 and 6 post-infection, which may indicate that *S. marcescens* can grow rapidly to overwhelm its host in the absence of AMPs.

FIG. 5: This is another great example of why it can be important to check protein levels. There are likely quite a few possible references for diet-induced changes to protein translation. One that comes to mind is the effect of diet on translational control through 4E-BP/eIF4E (e.g., Zid & Benzer in Cell 2009). Zid et al. suggested that increased 4E-BP activity inhibits eIF4E, which shifts translation to favor mRNAs with short, less structured 5'-UTR sequences. I don't recall whether immunity genes were looked at, but a quick check on flybase for the 6 AMPs in this figure show that they all have short 5' UTRs (9 to 74 bases). It's not clear whether the high sugar diets used in the current manuscript would be similar or not to the dietary restriction condition, but it might be worthwhile to look at 4E-BP phosphorylation or mentioning this connection between diet and translation in the Discussion.

We thank the reviewer for this thoughtful interpretation and for sharing the 4E-BP diet restriction paper, and both reviewers make valid points for a need to expand the connection between dietary sugar and protein levels on AMP translation. While measuring translation at genome scale or assessing the activity of proteins like 4E-BP are out of the scope of this project, we have added some relevant context to our discussion on lines 515-537 to speculate how diet-dependent effects on AMP levels could be mediated by nutrient sensing pathways such as insulin and TOR. We cite literature that demonstrates how Toll and IMD signaling suppress insulin activity to support secretion (Martinez, et al 2020, Plos Genetics 16:e1009192), which could potentially impact TOR dependent regulation of 4E-BP. We cite previous studies that speculate that insulin/TOR signaling and innate immune signaling may antagonize each other to regulate homeostasis depending on the organism’s physiological needs.

**ADDITIONAL COMMENTS**

The studies correlate sensitivity to infection with changes to several diet-induced phenotypes including AMP expression, bacterial proliferation, and internal carbohydrates. Although the interpretations are reasonable, it would have been more powerful to see that these associated changes are not observed in the Gram-positive bacteria that did not show diet-induced changes to survival. However, it would also be reasonable to fill in this gap in a subsequent study.

Additional characterization of the Gram-positive infections could add an interesting additional dimension to the paper. However, as explained in detail in response to an insightful comment by Reviewer 2 below, we have decided to minimize the emphasis on the Gram-positive infections in this paper. Briefly, we have evidence that infection dose might matter for the diet-sensitivity of those infections, but we do not presently have the capacity to repeat all the experiments over variable infection doses. As suggested by Reviewer 1, a thorough characterization of the effect of diet on Gram-positive infections and contrast to Gram-negative infections can be the subject of a future paper.

The authors' reasonably rule out an effect of fly-associated microbes on their phenotypes. However, I would be careful using the term 'gut microbiota' (line 142). It's more likely that the changes in diet are affecting the pool of environmental microbes, which is reflected in the number of bacteria seen in the fly gut. Studies that have examined this suggest that the fly gut usually reflects environmental microbes unless specific, strong (stable) bacterial colonizers are introduced, which do not seem that common in laboratory-maintained flies. To me, gut microbiota refers to something more specific than just bacteria from the environment that were ingested and passing through the gut.

We appreciate and agree with this comment about terminology of microbiological associations, especially in *Drosophila*. Since we did not explicitly measure establishment or persistence in the gut, instead of referring to “gut microbiota” in the manuscript, we know use the terms “microbiota” or “associated microbiota”. We also note in the text (lines 150-152) that varying diet affects the microbiological composition within the fly as well as in the rearing media.

Also, do infected flies pass live bacteria into the environment? If so, that provides another opportunity for Providencia or Serratia to interact with the medium. If these bacteria can access the environment, grow, and be reinfected (orally), that may be another path for affecting fly mortality. In this case, the studies in Fig. 4C-D might be better executed on the fly medium, rather than the minimal media used.

We thank the reviewer for asking this. Our lab and others routinely do experiments using the basic protocol described in our study, and we generally take on faith (supported by periodic control assays) that the infecting bacteria are not transmitted horizontally between hosts or disseminated through the housing environment. However, this comment prompted us to perform a more systematic analysis. Described below, the analysis confirms that our experimental procedures are robust and gives us confidence that our results are not artifacts deriving from transmission of bacteria within rearing vial. However, as these are not publication-quality experiments, we have decided not to include them in the manuscript or supplement.

We first tested whether infected flies could directly or indirectly pass bacteria to uninfected flies in a cohousing experiment. We infected flies with our standard procedure and placed them in a vial with uninfected flies (distinguished by a phenotypic marker) for either 1 or 4 days. We saw no mortality of uninfected flies during this window, and we observed that uninfected flies yield almost no colony forming units (CFUs) compared to infected flies or the initial inoculum dose given to the infected flies. As shown in the figure below, this is true for infections with both *Providencia* and *Serratia* infections.

Imagining that dead flies might spill an abundance of bacteria into the rearing medium, which might then proliferate and be acquired by uninfected bacteria, we tested whether inoculating the food with ~3x10^6^ cells of either pathogen could impact infection survival. As shown in the figure below, we saw no increase in mortality from flies maintained on food that had been inoculated with bacteria.

We understand that testing bacterial growth requirements in M9 minimal media is not equivalent to testing those requirements in the rearing environment. However, we do not have a good *in vitro* growth assay for bacteria in the *Drosophila* rearing media. Given the general lack of evidence for between-host transmission, we do not believe our observed diet effects on resistance to infection are artifacts of different rates of bacterial growth in the Drosophila rearing medium.

In Fig. 2D, the number of asterisks seems inconsistent with the p values given for Providencia and Serratia (2% vs. 16% in each).

We have corrected Fig. 2D to accurately reflect p-values in the figure legend. We thank the reviewer for bringing this to our attention.

**Reviewer 2:**

**Summary:**

 In this study, Darby et al. ask how dietary sugar alters the course of and response to infection. The authors explore this using six diets with increasing amounts of sugar (0-24%) and four pathogens (Gram-negative: P. rettgeri and S. marcescens; Gram-positive: E. faecalis and L. lactis). Interestingly, high sugar diets increase susceptibility to infection with Gram-negative bacteria but not Gram-positive bacteria. Additionally, a threshold effect is observed with increased lethality only occurring at 8% sucrose (P. rettgeri) or 16% (S. marcescens). The authors find that increased pathogen burden in flies on high sugar diets correlates with increased pathogen growth on media containing sugar (S. marcescens) and decreased production of the antimicrobial peptides cecropin and drosocin (P. rettgeri). This is an interesting, important study that begins to untangle the complex effects of diet on the response to infection, and the results show that distinct mechanisms underlie the effects of dietary sugar on infections with different pathogens. Some additional information would improve the study.

We are glad that the reviewer feels that the work is important and interesting and we have attempted to provide the additional information suggested. A detailed response to the reviewer’s comment’s is provided below.

**Major Issues: Key Experiments Required for Acceptance**

Three- to four-times fewer bacterial cells were injected into flies for the Gram-positive infections versus the Gram-negative infections. Given that the high sugar diet affected infection outcomes only in the Gram-negative groups, it seems possible that a higher initial bacterial dose for E. faecalis or L. lactis might reveal sugar-dependent effects. Have the authors tried this?

We thank the reviewer for this careful reading of the manuscript. The difference in inoculation dose between the Gram-positive versus Gram-negative infections presumably reflects small differences in the relationship between optical density and viable cell number under our growth conditions. In our previous experience, differences of this magnitude in inoculation dose can have small quantitative effects on timing of mortality and bacterial proliferation but generally do not have qualitative effects on major phenomena. Nevertheless, the reviewer’s comment prompted us to conduct a small experiment comparing infection with ~1000 cells (the inoculation in the submitted manuscript) to ~3000 cells (similar to the Gram-negative infections) of *E. faecalis* and *L. lactis* in flies fed 2% and 16% diets. Very much to our surprise, this difference in inoculation dose did alter the diet-dependence of the survivorship of infection of *E. faecalis* and we detected a small effect of diet on survivorship of *L. lactis* infection at both doses. These results are shown in the figure below.

Given the apparent complexity of the relationship between diet and response to the Gram-positive infections, we have elected to deemphasize the contrast between Gram-negative and Gram-positive infections in the manuscript. We have moved the Gram-positive survivorship plots that were formerly in Figure 1 to a supplemental figure S1. We draw attention to the difference in inoculation dose between the Gram-positive and Gram-negative infection experiments (line 138-144) and have added the graphs above as supplemental figure S1B and S1D.

In light of these new results, we have reduced our discussion of potential differences between the Toll and IMD pathways in their sensitivity to diet as a possible explanation for differences in survival of infection by alternative Gram-types (lines 439-441 We have added a statement that inoculation dose could explain the survival differences across the 0%-24% sucrose diet series and we then note that future investigations could focus on sugar-dependent effects of Gram-positive infection (lines 442-443), which could include the full complement of assays of diet-induced effects on immune phenotypes and carbohydrate stores at multiple inoculation doses (doing all of this would be well beyond the scope of the present manuscript). We removed the mention of Gram-positive infections from the abstract (line 26). We added more explicit rationale for using the specific Gram-negative (lines 121-124) and Gram-positive bacteria (lines 132-136) we tested, and also include additional rationale (lines 193-194) for our motivation to focus on *P. rettgeri* and *S. marcescens* for further infection experiments.

Figure 2A shows that uninfected flies consume about 60-70% as much of the high sucrose diets compared with low sucrose diets. How is intake of 2% versus 16% sucrose diet affected by infection? Knowing this would provide another layer of understanding the circulating and stored carbohydrate levels in panels B-D.

We had previously tried to measure feeding post-infection on low vs high sugar diets using our ExQ technique. However, this assay requires the flies to remain alive for the duration of the experiment to avoid conflating death with reduced feeding by live flies. Unfortunately, flies provided with high sugar diets exhibit high mortality within the first 24-hours of infection and we found it difficult to accurately quantify the amount of dye excreted by live flies within the sampling window. We therefore have low confidence in these data and we choose not to include post-infection feeding rates in the manuscript, despite agreeing with the reviewer that it is an interesting question.

Production of cecropin and drosocin is reduced by high sugar diet in the P. rettgeri infected animals, and therefore it would be expected that the 2% sucrose diet fed AMPdelta10 mutants have significantly worsened burden relative to wild type. Is this the case? For Figure 4A, how is the flattening effect of the AMPdelta10 mutation on the P. rettgeri bacterial burden achieved? Are CFUs on the 2% sucrose diet increased relative to wild type, are they reduced on the 16% sucrose diet, or some of both?

These questions are similar to a question raised by Reviewer 1 and a detailed response is given above. In brief, yes, the dAMP10 flies have higher burdens than wildtype flies, and we have added Supplementary Figure S4 to show this. The apparent flattening effect is an artifact of ascertaining only living flies. It arises as pathogen loads reach the maximum survivable burden. Flies that exceed this burden die (e.g., Duneau et al 2017 eLife.28298) and are consequently removed from the assay, resulting in an apparent plateau in the burden of living flies. We have clarified this in the manuscript on lines 289-291.

The units for AMP measurements (Figure 5C, 5D and 5F and Figure S3) are “Mr/fly”. What is “Mr”? It would be helpful, and valuable information for the field, to report the AMP results as mass (pg?)/fly instead. This should be possible based on the ELISA approach.

We have corrected the units to pg/fly in Fig. 5 and the Fig. S3. The *Mr* in the original versions was a relative molecular mass of the HA-FLAG-tagged-AMP compared to a standard peptide plotted on a standard curve. However, we acknowledge that this measure does not have much intuition, so we have added an equation to calculate the mass of produced AMPs to the Methods (line 717-727) and have added Supplementary Table 2 that includes the molecular masses of standard HA/FLAG peptide and the Drosocin-tagged and CecropinA1-tagged peptides.

The authors provide a cursory discussion of how dietary sugar might alter translation of antimicrobial peptides. Some previous work (Vasudevan et al. 2017, cited by the authors) delves into molecular mechanisms of translations of AMPs. How might dietary sugar impact such mechanisms?

As noted in response to a similar comment by Reviewer 1, we have added some relevant context to our discussion on lines 519-537 to speculate how diet-dependent effects on AMP levels could be mediated by direct or indirect effects on translation.

**ADDITIONAL COMMENTS**

The text has some grammatical errors. For example:
Line 188: “representative [of] the”
Line 377: “ELISA [to] quantify”
Line 390: “equivalent [in] flies”

Thank you for catching these grammatical errors. The specifically mentioned errors and a handful of others have been corrected.

The rationale for using mated females (line 531) should be provided.

We now provide rationale on lines 553-555 for using mated females. Mated is a more “natural” state than unmated given the rapidity with which *Drosophila* mate post-eclosion, mating will occur naturally in our experimental setup where males and females are cohoused after eclosion onto the experimental diets, and females are more likely than males to be immunologically sensitive to diet given their more demanding reproductive physiology.

The meaning of the black, purple and red font colors in the table sections of Figure 3 (“Proportion of flies alive at each time point) should be provided.

These different font colors indicate the proportion of surviving flies at the time of sampling: black 100%-80%, purple 79%-50%, red 50%-0%. We added this brief descriptor to the legend of figure 3.

For ELISA results in Figure 5, how many flies are homogenized per sample?

Each sample is a single fly. This point of methodology had been provided in the main body of the text (line 388) but was absent from the Methods section. We have now clarified in the Methods section (line 704 ) and in the Figure 5 legend (line 425-427).

Not all figure legends contain sample sizes. These should be included throughout the manuscript.

We have added sample sizes in the legends or directly to the plots for the following figures: Fig. S1, Fig. 2, Fig. S2, Fig. 3, Fig. S3, Fig. 4, Fig. S4, Fig. 5, Fig. S5, Fig. S6.
