# Supplementary material for: High sugar diets can increase susceptibility to bacterial infection in Drosophila melanogaster
Source: PLoS Pathog. 2024 Aug 12;20(8):e1012447. doi: 10.1371/journal.ppat.1012447 (PMC11341100; doi:10.1371/journal.ppat.1012447)
Supplement: Peer review history

# Author comment: Author response to previous submission

**DOI:** 10.1371/journal.ppat.1012447.r001

**Transfer Alert**

This paper was transferred from another journal. As a result, its full editorial history (including decision letters, peer reviews and author responses) may not be present.

17 Dec 2023

Attachment Submitted filename: Response_to_reviewer_15_dec_2023.pdf

# Peer review: Decision Letter 0

**DOI:** 10.1371/journal.ppat.1012447.r002

**Authors:** David S. Schneider, Matthew C. Wolfgang

29 Jan 2024

Dear Ms. Darby,

Thank you very much for submitting your manuscript "High sugar diets can increase susceptibility to bacterial infection in Drosophila melanogaster" for consideration at PLOS Pathogens. As with all papers reviewed by the journal, your manuscript was reviewed by members of the editorial board and by several independent reviewers. The reviewers appreciated the attention to an important topic. Based on the reviews, we are likely to accept this manuscript for publication, providing that you modify the manuscript according to the review recommendations.

Both reviewers were appreciative of the manuscript and had comments that might help improve its clarity.

Please prepare and submit your revised manuscript within 30 days. If you anticipate any delay, please let us know the expected resubmission date by replying to this email.

When you are ready to resubmit, please upload the following:

[1] A letter containing a detailed list of your responses to all review comments, and a description of the changes you have made in the manuscript.

Please note while forming your response, if your article is accepted, you may have the opportunity to make the peer review history publicly available. The record will include editor decision letters (with reviews) and your responses to reviewer comments. If eligible, we will contact you to opt in or out

[2] Two versions of the revised manuscript: one with either highlights or tracked changes denoting where the text has been changed; the other a clean version (uploaded as the manuscript file).

Important additional instructions are given below your reviewer comments.

Thank you again for your submission to our journal. We hope that our editorial process has been constructive so far, and we welcome your feedback at any time. Please don't hesitate to contact us if you have any questions or comments.

Sincerely,

David S. Schneider

Academic Editor

PLOS Pathogens

Matthew Wolfgang

Section Editor

PLOS Pathogens

Michael Malim

Editor-in-Chief

PLOS Pathogens

orcid.org/0000-0002-7699-2064

***********************

Both reviewers were appreciative of the manuscript and had comments that might help improve its clarity.

Reviewer Comments (if any, and for reference):

Reviewer's Responses to Questions

**Part I - Summary**

Please use this section to discuss strengths/weaknesses of study, novelty/significance, general execution and scholarship.

Reviewer #1: Darby et al. investigate the effect of high-sugar diets on susceptibility to bacterial infection in Drosophila. They survey multiple bacterial species to identify two Gram-negative bacteria that increase fly mortality on high-sugar diet, and further associate sensitivity to infection with diet-induced changes to immune gene expression (at the protein level) and bacterial proliferation. The manuscript is written clearly and conservatively, and the premise for the study in relation to previous work is well described. Although there is no simple and direct take-home message here--other than the idea that diet likely impacts both flies and bacteria to affect mortality--that is probably the nature of the complex interaction between host, pathogens, and diet. The experiments are fairly well designed and executed, and the results seem solid. I've highlighted a few questions and suggestions for further study. Although additional studies would add to the completeness of the paper, it would also be reasonable to leave most of these to a follow-up study.

Reviewer #2: In this study, Darby et al. ask how dietary sugar alters the course of and response to infection. The authors explore this using six diets with increasing amounts of sugar (0-24%) and four pathogens (Gram-negative: P. rettgeri and S. marcescens; Gram-positive: E. faecalis and L. lactis). Interestingly, high sugar diets increase susceptibility to infection with Gram-negative bacteria but not Gram-positive bacteria. Additionally, a threshold effect is observed with increased lethality only occurring at 8% sucrose (P. rettgeri) or 16% (S. marcescens). The authors find that increased pathogen burden in flies on high sugar diets correlates with increased pathogen growth on media containing sugar (S. marcescens) and decreased production of the antimicrobial peptides cecropin and drosocin (P. rettgeri). This is an interesting, important study that begins to untangle the complex effects of diet on the response to infection, and the results show that distinct mechanisms underlie the effects of dietary sugar on infections with different pathogens. Some additional information would improve the study.

**********

**Part II – Major Issues: Key Experiments Required for Acceptance**

Please use this section to detail the key new experiments or modifications of existing experiments that should be absolutely required to validate study conclusions.

Generally, there should be no more than 3 such required experiments or major modifications for a "Major Revision" recommendation. If more than 3 experiments are necessary to validate the study conclusions, then you are encouraged to recommend "Reject".

Reviewer #1: I have listed all of my comments in the "minor issues" section. The only key experiments from that list of suggestions that I would really want to see are additional, controlled Delta-AMP10 lifespan and bacteria proliferation studies to better support the stated interpretations. It would also be worth checking if infected flies are able to inoculate the fly food surface with the infecting bacteria, since this is potentially a major source for a diet-bacteria interaction.

Reviewer #2: 1. Three- to four-times fewer bacterial cells were injected into flies for the Gram-positive infections versus the Gram-negative infections. Given that the high sugar diet affected infection outcomes only in the Gram-negative groups, it seems possible that a higher initial bacterial dose for E. faecalis or L. lactis might reveal sugar-dependent effects. Have the authors tried this?

2. Figure 2A shows that uninfected flies consume about 60-70% as much of the high sucrose diets compared with low sucrose diets. How is intake of 2% versus 16% sucrose diet affected by infection? Knowing this would provide another layer of understanding the circulating and stored carbohydrate levels in panels B-D.

3. Production of cecropin and drosocin is reduced by high sugar diet in the P. rettgeri infected animals, and therefore it would be expected that the 2% sucrose diet fed AMPdelta10 mutants have significantly worsened burden relative to wild type. Is this the case? For Figure 4A, how is the flattening effect of the AMPdelta10 mutation on the P. rettgeri bacterial burden achieved? Are CFUs on the 2% sucrose diet increased relative to wild type, are they reduced on the 16% sucrose diet, or some of both?

4. The units for AMP measurements (Figure 5C, 5D and 5F and Figure S3) are “Mr/fly”. What is “Mr”? It would be helpful, and valuable information for the field, to report the AMP results as mass (pg?)/fly instead. This should be possible based on the ELISA approach.

5. The authors provide a cursory discussion of how dietary sugar might alter translation of antimicrobial peptides. Some previous work (Vasudevan et al. 2017, cited by the authors) delves into molecular mechanisms of translations of AMPs. How might dietary sugar impact such mechanisms?

**********

**Part III – Minor Issues: Editorial and Data Presentation Modifications**

Please use this section for editorial suggestions as well as relatively minor modifications of existing data that would enhance clarity.

Reviewer #1: METHODS: The experiments are generally clear and well designed. My greatest (still minor) concern is why the sucrose concentration series is provided immediately upon eclosion. It was my understanding that the first couple of days of adulthood (and especially the first hours post-eclosion) is still a period with some ongoing 'development' or developmental plasticity. Many studies avoid using very young adults for behavioral and other assays for this reason. I would have preferred maintaining flies on a standard diet until at least 2 days of age before starting the sucrose series. I don't think it's a huge deal here, but the authors could discuss this caveat or replicate an experiment or two with older flies.

FIG. 2: Looking at the consumption data, it seems that the difference in feeding would result in a ~400% increase in sucrose ingestion between the 2% and 16% sucrose diets used. While I agree that this is the 'bigger' change, it should be noted that protein ingestion is also reduced by about 30-35%. Thus, it is not clear if phenotypes (especially changes in AMP production) are due to more sugar, less protein, or both.

FIG. 4: The use of the Delta-AMP10 line is a great idea, but the results here are weaker. In Fig. 4A, diet does not affect P. rettgeri proliferation in the fly mutant. But only time points up to 12 hours are shown. In Fig. 3A, there is also no difference in P. rettgeri proliferation in Canton-S until 12 hours and later. It would thus be better to include later time points for the Delta-AMP10 study (as well as its genetic background control assayed in the same trial). The results for S. marcescens are even more challenging to interpret, since 16% sugar seems to consistently reduce bacterial load early, then increase it later, but only for a few hours, after which they're equivalent until much later. I agree though that, at least early in the infection, the effect of diet on S. marcescens seems reasonably consistent between genotypes. Also, what does survival look like in Delta-AMP10 upon infection? That could go a long way in supporting some of the interpretations of the proliferation data.

FIG. 5: This is another great example of why it can be important to check protein levels. There are likely quite a few possible references for diet-induced changes to protein translation. One that comes to mind is the effect of diet on translational control through 4E-BP/eIF4E (e.g., Zid & Benzer in Cell 2009). Zid et al. suggested that increased 4E-BP activity inhibits eIF4E, which shifts translation to favor mRNAs with short, less structured 5'-UTR sequences. I don't recall whether immunity genes were looked at, but a quick check on flybase for the 6 AMPs in this figure show that they all have short 5' UTRs (9 to 74 bases). It's not clear whether the high sugar diets used in the current manuscript would be similar or not to the dietary restriction condition, but it might be worthwhile to look at 4E-BP phosphorylation or mentioning this connection between diet and translation in the Discussion.

ADDITIONAL COMMENTS:

The studies correlate sensitivity to infection with changes to several diet-induced phenotypes including AMP expression, bacterial proliferation, and internal carbohydrates. Although the interpretations are reasonable, it would have been more powerful to see that these associated changes are not observed in the Gram-positive bacteria that did not show diet-induced changes to survival. However, it would also be reasonable to fill in this gap in a subsequent study.

The authors' reasonably rule out an effect of fly-associated microbes on their phenotypes. However, I would be careful using the term 'gut microbiota' (line 142). It's more likely that the changes in diet are affecting the pool of environmental microbes, which is reflected in the number of bacteria seen in the fly gut. Studies that have examined this suggest that the fly gut usually reflects environmental microbes unless specific, strong (stable) bacterial colonizers are introduced, which do not seem that common in laboratory-maintained flies. To me, gut microbiota refers to something more specific than just bacteria from the environment that were ingested and passing through the gut.

Also, do infected flies pass live bacteria into the environment? If so, that provides another opportunity for Providencia or Serratia to interact with the medium. If these bacteria can access the environment, grow, and be reinfected (orally), that may be another path for affecting fly mortality. In this case, the studies in Fig. 4C-D might be better executed on the fly medium, rather than the minimal media used.

In Fig. 2D, the number of asterisks seems inconsistent with the p values given for Providencia and Serratia (2% vs. 16% in each).

Reviewer #2: 1. The text has some grammatical errors. For example:

Line 188: “representative [of] the”

Line 377: “ELISA [to] quantify”

Line 390: “equivalent [in] flies”

2. The rationale for using mated females (line 531) should be provided.

3. The meaning of the black, purple and red font colors in the table sections of Figure 3 (“Proportion of flies alive at each time point) should be provided.

4. For ELISA results in Figure 5, how many flies are homogenized per sample?

5. Not all figure legends contain sample sizes. These should be included throughout the manuscript.

**********

PLOS authors have the option to publish the peer review history of their article (what does this mean?). If published, this will include your full peer review and any attached files.

If you choose “no”, your identity will remain anonymous but your review may still be made public.

**Do you want your identity to be public for this peer review?** For information about this choice, including consent withdrawal, please see our Privacy Policy.

Reviewer #1: No

Reviewer #2: No

Figure Files:

While revising your submission, please upload your figure files to the Preflight Analysis and Conversion Engine (PACE) digital diagnostic tool, https://pacev2.apexcovantage.com. PACE helps ensure that figures meet PLOS requirements. To use PACE, you must first register as a user. Then, login and navigate to the UPLOAD tab, where you will find detailed instructions on how to use the tool. If you encounter any issues or have any questions when using PACE, please email us at figures@plos.org.

Data Requirements:

Please note that, as a condition of publication, PLOS' data policy requires that you make available all data used to draw the conclusions outlined in your manuscript. Data must be deposited in an appropriate repository, included within the body of the manuscript, or uploaded as supporting information. This includes all numerical values that were used to generate graphs, histograms etc.. For an example see here: http://www.plosbiology.org/article/info%3Adoi%2F10.1371%2Fjournal.pbio.1001908#s5.

Reproducibility:

To enhance the reproducibility of your results, we recommend that you deposit your laboratory protocols in protocols.io, where a protocol can be assigned its own identifier (DOI) such that it can be cited independently in the future. Additionally, PLOS ONE offers an option to publish peer-reviewed clinical study protocols. Read more information on sharing protocols at https://plos.org/protocols?utm_medium=editorial-email&utm_source=authorletters&utm_campaign=protocols

References:

Please review your reference list to ensure that it is complete and correct. If you have cited papers that have been retracted, please include the rationale for doing so in the manuscript text, or remove these references and replace them with relevant current references. Any changes to the reference list should be mentioned in the rebuttal letter that accompanies your revised manuscript. If you need to cite a retracted article, indicate the article’s retracted status in the References list and also include a citation and full reference for the retraction notice.

# Author comment: Author response to Decision Letter 0

**DOI:** 10.1371/journal.ppat.1012447.r003

29 Apr 2024

Attachment Submitted filename: ReviewerResponses_resubmission.docx

# Peer review: Decision Letter 1

**DOI:** 10.1371/journal.ppat.1012447.r004

**Authors:** Matthew C. Wolfgang

23 Jul 2024

Dear Ms. Darby,

We are pleased to inform you that your manuscript 'High sugar diets can increase susceptibility to bacterial infection in Drosophila melanogaster' has been provisionally accepted for publication in PLOS Pathogens.

Before your manuscript can be formally accepted you will need to complete some formatting changes, which you will receive in a follow up email. A member of our team will be in touch with a set of requests.

Please note that your manuscript will not be scheduled for publication until you have made the required changes, so a swift response is appreciated.

IMPORTANT: The editorial review process is now complete. PLOS will only permit corrections to spelling, formatting or significant scientific errors from this point onwards. Requests for major changes, or any which affect the scientific understanding of your work, will cause delays to the publication date of your manuscript.

Should you, your institution's press office or the journal office choose to press release your paper, you will automatically be opted out of early publication. We ask that you notify us now if you or your institution is planning to press release the article. All press must be co-ordinated with PLOS.

Thank you again for supporting Open Access publishing; we are looking forward to publishing your work in PLOS Pathogens.

Best regards,

Matthew C. Wolfgang, Ph.D.

Section Editor

PLOS Pathogens

Matthew Wolfgang

Section Editor

PLOS Pathogens

Michael Malim

Editor-in-Chief

PLOS Pathogens

orcid.org/0000-0002-7699-2064

***********************************************************

Reviewer Comments (if any, and for reference):

Reviewer's Responses to Questions

**Part I - Summary**

Please use this section to discuss strengths/weaknesses of study, novelty/significance, general execution and scholarship.

Reviewer #1: The revised manuscript addresses all of the previous concerns. I have no further suggestions.

Reviewer #2: The authors' revisions, both in terms of changes to the text as well as new experimental results, have improved the manuscript. This study is a valuable contribution to the field.

**********

**Part II – Major Issues: Key Experiments Required for Acceptance**

Please use this section to detail the key new experiments or modifications of existing experiments that should be absolutely required to validate study conclusions.

Generally, there should be no more than 3 such required experiments or major modifications for a "Major Revision" recommendation. If more than 3 experiments are necessary to validate the study conclusions, then you are encouraged to recommend "Reject".

Reviewer #1: None

Reviewer #2: none noted

**********

**Part III – Minor Issues: Editorial and Data Presentation Modifications**

Please use this section for editorial suggestions as well as relatively minor modifications of existing data that would enhance clarity.

Reviewer #1: None

Reviewer #2: none noted

**********

PLOS authors have the option to publish the peer review history of their article (what does this mean?). If published, this will include your full peer review and any attached files.

If you choose “no”, your identity will remain anonymous but your review may still be made public.

**Do you want your identity to be public for this peer review?** For information about this choice, including consent withdrawal, please see our Privacy Policy.

Reviewer #1: No

Reviewer #2: No

# Editor report: Acceptance letter

**DOI:** 10.1371/journal.ppat.1012447.r005

**Authors:** Matthew C. Wolfgang

6 Aug 2024

Dear Dr. Darby,

We are delighted to inform you that your manuscript, "High sugar diets can increase susceptibility to bacterial infection in Drosophila melanogaster," has been formally accepted for publication in PLOS Pathogens.

We have now passed your article onto the PLOS Production Department who will complete the rest of the pre-publication process. All authors will receive a confirmation email upon publication.

The corresponding author will soon be receiving a typeset proof for review, to ensure errors have not been introduced during production. Please review the PDF proof of your manuscript carefully, as this is the last chance to correct any scientific or type-setting errors. Please note that major changes, or those which affect the scientific understanding of the work, will likely cause delays to the publication date of your manuscript. Note: Proofs for Front Matter articles (Pearls, Reviews, Opinions, etc...) are generated on a different schedule and may not be made available as quickly.

Soon after your final files are uploaded, the early version of your manuscript, if you opted to have an early version of your article, will be published online. The date of the early version will be your article's publication date. The final article will be published to the same URL, and all versions of the paper will be accessible to readers.

Thank you again for supporting open-access publishing; we are looking forward to publishing your work in PLOS Pathogens. 

Best regards,

Michael Malim

Editor-in-Chief

PLOS Pathogens

orcid.org/0000-0002-7699-2064
